# Diet Composition of the Wild Stump-Tailed Macaque (*Macaca arctoides*) in Perlis State Park, Peninsular Malaysia, Using a Chloroplast tRNL DNA Metabarcoding Approach: A Preliminary Study

**DOI:** 10.3390/ani10122215

**Published:** 2020-11-26

**Authors:** Nur Azimah Osman, Muhammad Abu Bakar Abdul-Latiff, Abd Rahman Mohd-Ridwan, Salmah Yaakop, Shukor Md Nor, Badrul Munir Md-Zain

**Affiliations:** 1 Department of Biological Sciences and Biotechnology, Faculty of Science and Technology Universiti Kebangsaan Malaysia, Bangi 43600, Selangor, Malaysia; azimahosman@gmail.com (N.A.O.); ridwanrahman2@gmail.com (A.R.M.-R.); salmah78@ukm.edu.my (S.Y.); shukor@ukm.edu.my (S.M.N.); 2 School of Biology, Faculty of Applied Sciences, Universiti Teknologi Mara Negeri Sembilan, Kuala Pilah 72000, Negeri Sembilan, Malaysia; 3 Oasis Integrated Group (OIG), Institute for Integrated Engineering (I²E), Universiti Tun Hussein Onn Malaysia, Parit Raja 86400, Johor, Malaysia; latiff@uthm.edu.my; 4 Faculty of Applied Sciences and Technology, Universiti Tun Hussein Onn Malaysia (Pagoh Campus), Muar 84000, Johor, Malaysia; 5 Centre for Pre-University Studies, Universiti Malaysia Sarawak, Kota Samarahan 94300, Sarawak, Malaysia

**Keywords:** Malaysian primates, *Macaca arctoides*, plant metabarcoding, tRNL

## Abstract

**Simple Summary:**

This study investigated plant diet of wild *Macaca arctoides* in the Malaysia–Thailand border region using a chloroplast tRNL DNA metabarcoding approach. It is a comprehensive molecular technique to assess foods eaten by primates. We have chosen chloroplast tRNL because this region has been widely used for identifying plant species. Chloroplast tRNL DNA was amplified and sequenced using the Illumina MiniSeq platform. Sequences were analyzed using the CLC Genomic Workbench software version 12.0 to check for *M. arctoides* plant diet. Across these samples, we successfully identified 29 plant orders, 46 families, 124 genera, and 145 species. As the first report in Malaysia, the findings provide an important understanding on diet of wild *M. arctoides* that only reside in Perlis State Park, Malaysia.

**Abstract:**

Understanding dietary diversity is a fundamental task in the study of stump-tailed macaque, *Macaca arctoides* in its natural habitat. However, direct feeding observation and morphological identification using fecal samples are not effective and nearly impossible to obtain in natural habitats because this species is sensitive to human presence. As ecological methods are challenging and time-consuming, DNA metabarcoding offers a more powerful assessment of the diet. We used a chloroplast tRNL DNA metabarcoding approach to identify the diversity of plants consumed by free-ranging *M. arctoides* in the Malaysia–Thailand border region located in Perlis State Park, Peninsular Malaysia. DNA was extracted from three fecal samples, and chloroplast tRNL DNA was amplified and sequenced using the Illumina MiniSeq platform. Sequences were analyzed using the CLC Genomic Workbench software. A total of 145 plant species from 46 families were successfully identified as being consumed by *M. arctoides*. The most abundant species were yellow saraca, *Saraca thaipingensis* (11.70%), common fig, *Ficus carica* (9.33%), aramata, *Clathrotropis brachypetala* (5.90%), sea fig, *Ficus superba* (5.44%), and envireira, *Malmea dielsiana* (1.70%). However, *Clathrotropis* and *Malmea* are not considered Malaysian trees because of limited data available from Malaysian plant DNA. Our study is the first to identify plant taxa up to the species level consumed by stump-tailed macaques based on a DNA metabarcoding approach. This result provides an important understanding on diet of wild *M. arctoides* that only reside in Perlis State Park, Malaysia.

## 1. Introduction

*Macaca arctoides* also known as the stump-tailed macaque or bear macaque (Geoffroy 1831), is widely distributed throughout South Asia (India, Bangladesh), Southwestern China, and Southeast Asia (Myanmar, Bangladesh, Thailand, Laos, Cambodia, Vietnam, and Malaysia) [1]. In Malaysia, the stump-tailed macaque is locally known as beruk kentoi and only inhabits the Perlis State Park (PSP) in the northern region of Peninsular Malaysia [2,3]. There have been few studies determining how the macaque interacts and thrives in this habitat. PSP is located on the western border between Perlis State and Thailand and includes the Mata Ayer Forest Reserve and the Wang Mu Forest Reserve which encompasses a total area of 5015 ha [4]. It is also located within the Nakawan Range, the longest continuous range of limestone hills in Malaysia [5]. PSP shares a unique distribution of flora and fauna with the Thaleban National Park in southern Thailand signifying both as prominent transboundary international parks [6]. The population of wild *M. arctoides* in Malaysia is threatened due to illegal hunting in PSP [3].

The availability and distribution of food resources are important factors in determining the ecological variations of primate species [7]. In natural habitats, primates consume certain parts of over 100 plant species including buds, fruits, young leaves, bark, roots, and flowers [8,9]. However, the diet of *M. arctoides* remains largely unknown. Indeed, more foraging and dietary studies have been done on long-tailed macaque and pig-tailed macaque. For example, Sha and Hanya [10] determined the natural foods consumed by the long-tailed macaque (*Macaca fascicularis*) which included 17 genera of plants in Singapore. *Axonopus compressus* (grass), *Villebrunea rubescens* (daun jilat), and *Caryota mitis* (fishtail palm) were the three most frequent plant species consumed by *M. fascicularis* in Telaga Warna, Bogor, West Java Indonesia [11]. In addition, the five most frequent plant species consumed by *M. fascicularis* were oil palm, *Elaeis guineensis* (11%), fig, *Ficus variegate* (4%), senduduk, *Melastoma malabathricum* (3.1%), bamboo, *Schizostachyum jaculans* (3.1%), and brown salwood, *Acacia mangium* (2.7%) in a mixed landscape consisting of urban, agro-forested areas and forest fragments in Malaysia [12]. Another macaque group in Peninsular Malaysia, pig-tailed macaque (*Macaca nemestrina*), was observed to consume oil palm parts, including attached and fallen oil palm fruits, seeds, and flowers [13].

DNA metabarcoding is used to identify organisms from a sample containing DNA materials. Taberlet et al. [14] introduced the term ‘DNA metabarcoding’ for the identification of multiple species using total or often degraded DNA extracted from bulk samples of entire organisms or from an environmental sample. Taberlet et al. [15] designed the oligonucleotide primer used for the identification of shorter fragments of the chloroplast tRNL (UAA) intron. These regions are highly conserved, and the amplification system is very robust for plant species identification [16]. Metabarcoding using tRNL produced thousands of reads consistent with the known diet of the red-shanked douc langurs (*Pygathrix nemaeus*); however, the barcodes were too short to identify several plant species within a genus [17]. This technique revealed at least 53 plant species from 33 families in a dietary profile of an endangered population of the banded leaf monkey (*Presbytis femoralis*) [18]. In addition, tRNL yielded greater numbers of sequences with similar sequencing effort, and a higher resolution of taxonomic identification in dietary studies of wild white-faced capuchins (*Cebus capucinus*) [19].

A recent field observation study of the Malaysian stump-tailed macaques indicated that they were only found in the Wang Kelian forest areas of PSP [3]. All individuals were counted in two free-ranging groups and the natural habitat may significantly influence their diet. Because no information is available regarding the natural food consumed by wild *M. arctoides*, we investigated the plant diversity consumed by *M. arctoides* in the Malaysia–Thailand border region using the chloroplast tRNL DNA metabarcoding approach. A clear understanding of the natural diet of this macaque will lead to the more effective conservation management of this species.

## 2. Materials and Methods

### 2.1. Fecal Sample Collection

**Figure 1:**
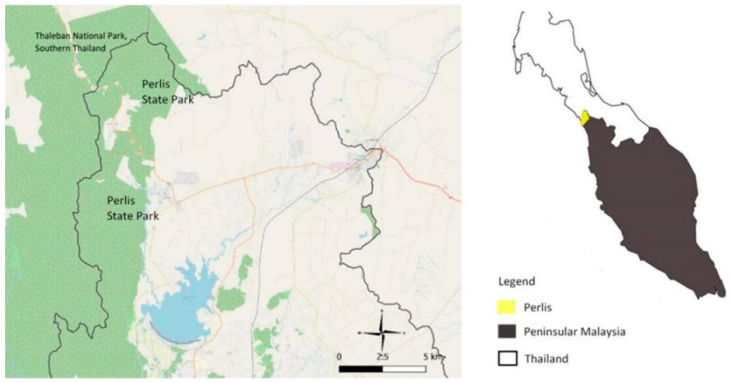
Map of Perlis State Park, Perlis Malaysia.

**Figure 2:**
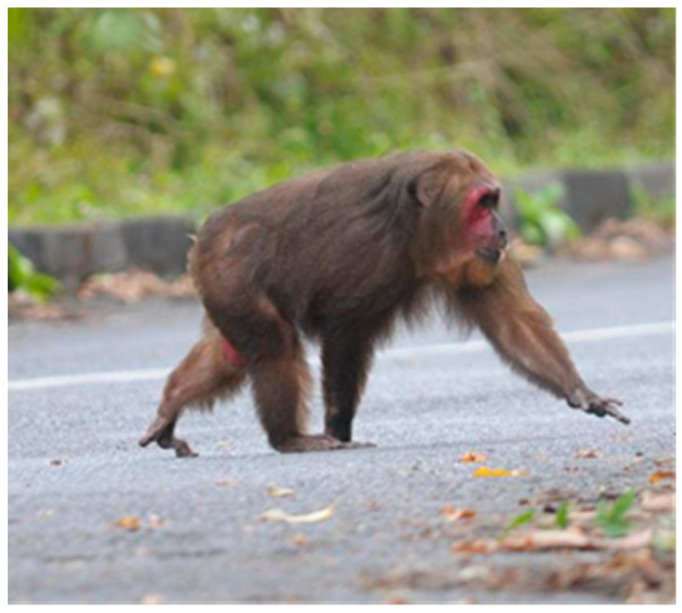
The free-ranging stump-tailed macaque in Wang Kelian, Perlis State Park, Malaysia [3].

All analyzed samples (*n* = 3) in the present study were collected from non-invasive fecal material sampled in February 2019 during the dry season at the Wang Kelian forest areas of PSP in order to restrict physical contact and potential disturbance to wild stump-tailed macaques (Figure 1). This free-ranging species can be found primarily at PSP in Malaysia (Figure 2). Intensive field observations with scanning sampling were conducted from September 2018 to January 2019 [3]. The number of fecal samples was relatively low because this species is hard to find and extremely sensitive to human presence. These macaque groups frequently disappear for several days before reappearing in the observation area [3]. We considered the specific area of observing *M. arctoides* at PSP based on an intensive field survey conducted by Syamil et al. [3]. We conducted the observation on that area and waited for macaques to drop the fecal. We collected the fecal once stump-tailed macaques left the area. The fresh fecal samples were also identified through the physical character of the fecal. The fecal samples were collected in sterile 15 mL tubes and fixed in 95% ethanol for long-term storage [20,21]. The three samples were labelled MA15, MA2, and MA14, and stored at −20 °C. Stump-tailed macaque genetic identification was performed using phylogenetic analysis of mitochondrial DNA D-loop region sequences. Maximum Parsimony (MP) phylogenetic tree formed stump-tailed macaque clade with 95% bootstrap confidence (data not published).

### 2.2. DNA Extraction

DNA was extracted from approximately 400 mg of each fecal sample using the innuPREP Stool DNA Kit (Analytik Jena, Jena, Germany). For each extraction, the surface and interior of the fecal pellet were sampled [17]. DNA integrity was examined by gel electrophoresis and the DNA concentration was measured by spectrophotometric and fluorometric methods using Implen NanoPhotometer and a Qubit 4.0 dsDNA HS Assay Kit (Life Technologies, Carlsbad, CA, USA). Samples were labelled and stored at −20 °C.

### 2.3. PCR Amplification

PCR amplification was carried out using the previously described primers targeting the P6 loop of the tRNL intron [16]. This region was amplified using the following primers: gtRNL For (5′-TCG TCG GCA GCG TCA GAT GTG TAT AAG AGA CAG GGG CAA TCC TGA GCC AA-3′) and hTRNL Rev (5′-GTC TCG TGG GCT CGG AGA TGT GTA TAA GAG ACA GCC ATT GAG TCT CTG CAC CTA TC-3′). All amplification reactions were done in a 25 μL total volume. The PCR mixture consisted of 12.5 μL of 2× KAPA HiFi HotStart Ready Mix (KAPA Biosystems, Wilmington, MA, USA), 5 μL for each of forward and reverse primer (10 μM), and 2.5 μL of template DNA in a final volume of 25 μL. The amplification reactions were performed on an AlphaTM PCRmax Alpha Cycler using the following program: 95 °C for 3 min, followed by 35 cycles of denaturation at 95 °C for 30 s, annealing at 55 °C for 30 s, extension at 72 °C for 30 s, and a final extension step at 72 °C for 5 min. The PCR products were then purified using Kapa pure beads (KAPA Biosystems, Wilmington, MA, USA). The purified products underwent a second PCR reaction using the Nextera XT Index Kit V2 (Illumina Inc., San Diego, CA, USA). The PCR reaction consisted of 5 µL for each of the Index 1 and Index 2 primers, 12.5 µL of 2× KAPA HiFi HotStart Ready Mix, and 2.5 µL of purified DNA. The amplification reactions were performed as follows: 95 °C for 30 s, followed by 12 cycles of 95 °C for 10 s, 55 °C for 30 s and 72 °C for 30 s, and a final extension step at 72 °C for 5 min. LabChip^®^ GX Touch 24 (Waltham, MA, USA) were used to verify PCR product size after visualizing with ultraviolet light.

### 2.4. Illumina MiniSeq-DNA Sequencing of tRNL Gene

Samples were individually indexed, using a barcode for the tRNL amplicons of each sample. The amplicons were amplified and quantified using quantitative polymerase chain reaction (qPCR). The reaction was carried out using the KAPA SYBR^®^ FAST qPCR Master Mix (KAPA Biosystems, Wilmington, MA, USA). The PCR mixture contained 10 µL KAPA SYBR^®^ FAST qPCR Master Mix, 2 µL of primer mix, 4 µL of indexed-amplicon, and 4 µL of RNase-free distilled water in a final volume of 20 µL. The PCR reactions were performed on a PCRmax Eco 48 real-time PCR system using the following program: 95 °C for 5 min, followed by 25 cycles of 95 °C for 40 s, 60 °C for 2 min, 72 °C for 1 min, and a final extension step at 72 °C for 7 min. To obtain a similar number of reads for each sample, normalization of the concentration of each sample is vital for the success of DNA sequencing. The amplicons were normalized based on data generated by qPCR and Qubit quantification. The indexed amplicons were then pooled into a single library for next-generation sequencing. The PhiX Control Kit was spiked in together with the normalized library as a control for the sequencing reactions. The final library was paired end sequenced at 2 × 150 bp using a MiniSeq High Throughput Reagent Kit on the Illumina MiniSeq platform (Illumina Inc., San Diego, CA, USA). Sequencing was done at the Evolutionary and Conservation Genetic Laboratory in the Department of Technology and Natural Resources, Universiti Tun Hussein Onn (UTHM), Malaysia.

### 2.5. Statistical Analysis

The quality filtering and demultiplexing of the resulting sequences were conducted using the CLC Genomic Workbench software version 12.0 (CLC) (Qiagen, Hilden, Germany). An initial assessment of the quality scores for the Illumina data was done using FASTQ file. The operational taxonomical units (OTUs) were clustered at 97% similarity and represented by a single sequence. The OTUs were aligned using the MUSCLE tool in CLC. Rarefaction curves were then plotted with the number of OTUs observed with a given sequencing depth using CLC. The alpha diversity used to assess plant species richness in the stump-tailed macaque was generated using the PAST software version 4.03 [22]. The relationship between the samples was displayed by principal coordinates analysis (PCoA) using the CLC software. Diversity *t*-test was conducted to compare the abundance of plants consumed by these stump-tailed macaques using the PAST 3 software. A Venn diagram was generated to determine the shared and unique OTUs among the three stump-tailed macaques at 97% similarity. To assess the relationship of the plant species community among the three samples, a phylogenetic dendrogram and heatmap were constructed with 1000 bootstraps following the Bray–Curtis distance. Statistical significance was set at *p* < 0.05.

## 3. Results

### 3.1. NGS Data Analysis and Sequence Filtering

We confirmed that the concentration of the DNA extracts ranged from 10.4 ng/µL to 15.40 ng/µL. We measured the quantity of DNA by qPCR to be 1.40 pM to 1.56 pM. As our study examined free-ranging wild stump-tailed macaque populations, we did not have direct observation information on diet. Therefore, we used the fecal metabarcoding approach to assess plant consumption from three representative samples in PSP.

The tRNL intron sequence of representative wild *M. arctoides* in PSP was successfully amplified. The Illumina next-generation sequencing produced 1,013,236 reads, ranging from 138,004 to 208,521 OTUs sequences and corresponding to 86,111 unique sequences. The final dataset was obtained by sequence filtering to exclude low-quality sequence reads, chimera, and subsequently OTU clustering. At the 97% similarity cut-off, 501,474 OTUs were generated with the highest OTUs is MA15 (208,521) followed by MA2 (154,949) and MA14 (138,004).

### 3.2. Diet Richness and Composition of the Wild M. Arctoides in PSP

The tRNL intron sequence of the representative wild *M. arctoides* samples were successfully amplified. Across these samples, we successfully identified 29 plant orders, 46 families, 124 genera, and 145 species (Table 1).

**Figure 3:**
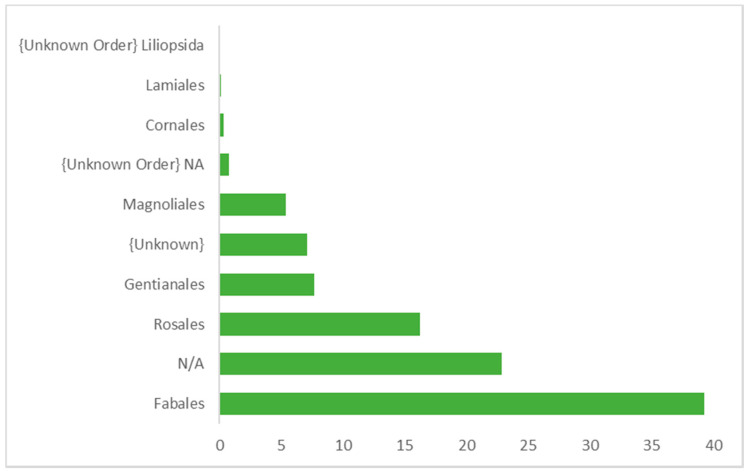
Order level distribution (%) in the diverse plants consumed by wild *Macaca arctoides* in Perlis State Park (PSP).

**Figure 4:**
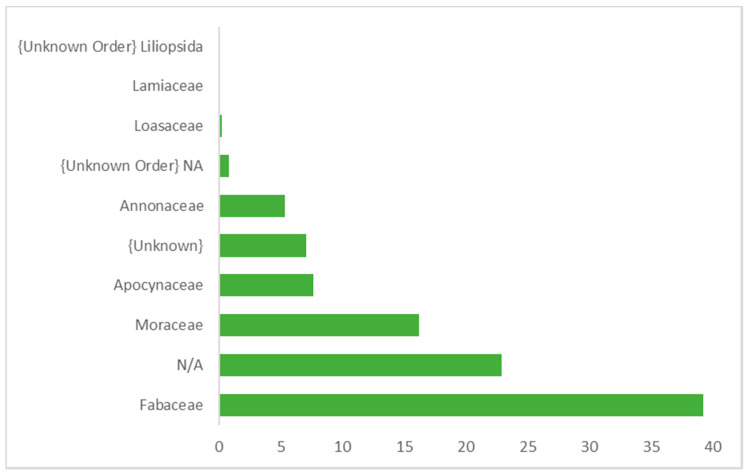
Family level distribution (%) in the diverse plants consumed by wild *M. arctoides* in PSP.

**Figure 5:**
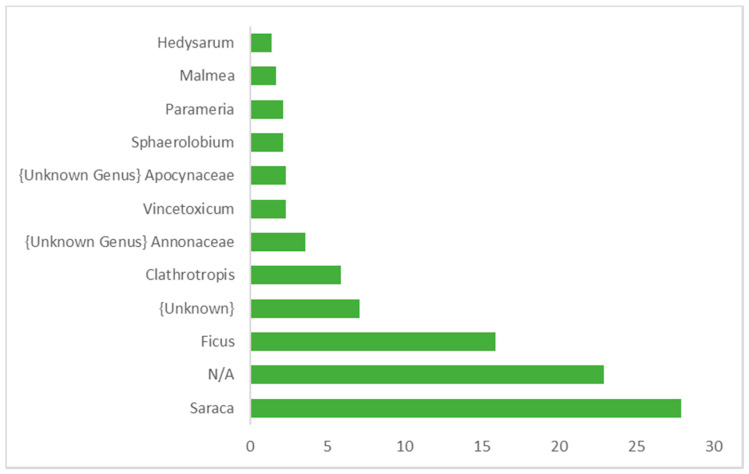
Genus level distribution (%) in the diverse plants consumed by wild *M. arctoides* in PSP.

Figure 3, Figure 4 and Figure 5 show the relative abundance of plant order, family, and genus (over 1% abundance) consumed by wild *M. arctoides*. Plants from the order Fabales were the most represented order. Other orders including Rosales, Gentianales, Magnoliales, Cornales, Lamiales, and four unknown orders were also identified. Fabaceae was the plant family accounting for the highest relative abundance (Figure 4). Sequences assigned to Moraceae and Apocynaceae were also among the most frequently occurring plant families. The most represented plant genera consumed by wild *M. arctoides* were *Saraca* (yellow saraca), *Ficus* (fig), *Clathrotropis* (aramata), and two unknown genera (Figure 5).

The tRNL sequences accounting for the highest abundance of plant species consumed by wild *M. arctoides* in PSP were yellow saraca, *Saraca thaipingensis* (11.70%), common fig, *Ficus carica* (9.33%), aramata, *Clathrotropis brachypetala* (5.90%), sea fig, *Ficus superba* (5.44%), and envireira, *Malmea dielsiana* (1.70%).

### 3.3. Heatmap, PCoA, Diversity Indices, and Venn Diagram

The alpha diversity indices (Chao-1, Simpson, Shannon) of overall OTUs observed in PSP indicated that *M. arctoides* has a highly diverse plant diet (Table 2). The Evenness index showed that the plant species consumed by this macaque is uniformly distributed. Based on the Shannon index, stump-tailed macaque of MA15 showed slightly higher results in terms of plant richness and evenness (*H* = 8.106) followed by MA2 (*H* = 7.387) and MA14 (*H* = 7.288). Similarly, the Simpson index showed the same pattern of the value. In contrast, MA2 is significantly higher in Chao 1 richness estimator which is 40,080 followed by MA14 (38,610) and MA15 (25,210).

**Figure 6:**
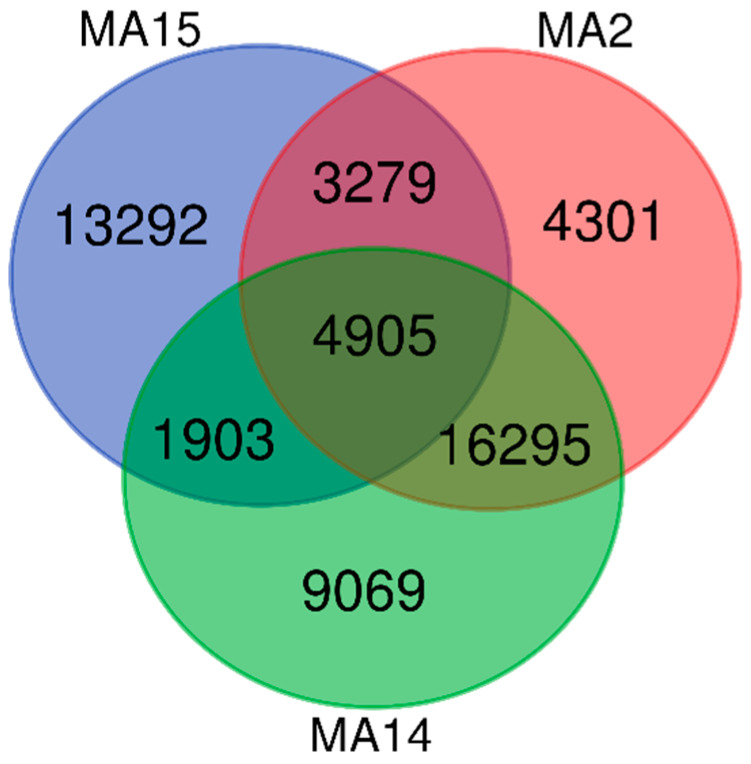
The Venn diagram illustrated the number of shared operational taxonomical units (OTUs) between the three representatives of *M. arctoides* at the 97% similarity. Colored circles represent each individual, and intersection part between circles represent the number of shared OTUs.

The resulting Venn diagram demonstrated an overlap between representative samples of wild *M. arctoides* inhabiting PSP (Figure 6). MA15 has the most unique sequences (13,292 OTUs) as compared to MA14 (9069 OTUs) and MA2 (4301 OTUs). The number of OTUs shared by these three individuals was 4905.

**Figure 7:**
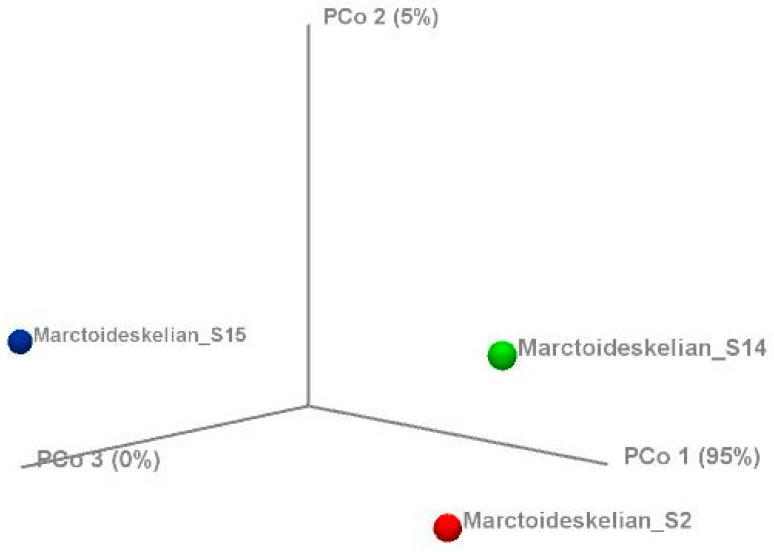
A three-dimensional plot of weighted UniFrac-based principal coordinates analysis (PCoA). The plot was created using the pairwise weighted UniFrac distances (PCo1 variability at 95%, PCo2 variability at 5%, and PCo3 variability at 0%).

Relationships between the representative samples were evaluated using UniFrac-based principal coordinates analysis (PCoA) (Figure 7). The plot was created using the pairwise weighted UniFrac distances accounting for diet richness and composition (PCo1 variability at 95%, PCo2 variability at 5%, and PCo3 variability at 0%). The representative samples of wild *M. arctoides* revealed the close relationship between individuals MA2 and MA14. Further analysis through a *t*-test has revealed a significant difference in the plant diversity consumed by these two individuals (*p* < 0.05).

**Figure 8:**
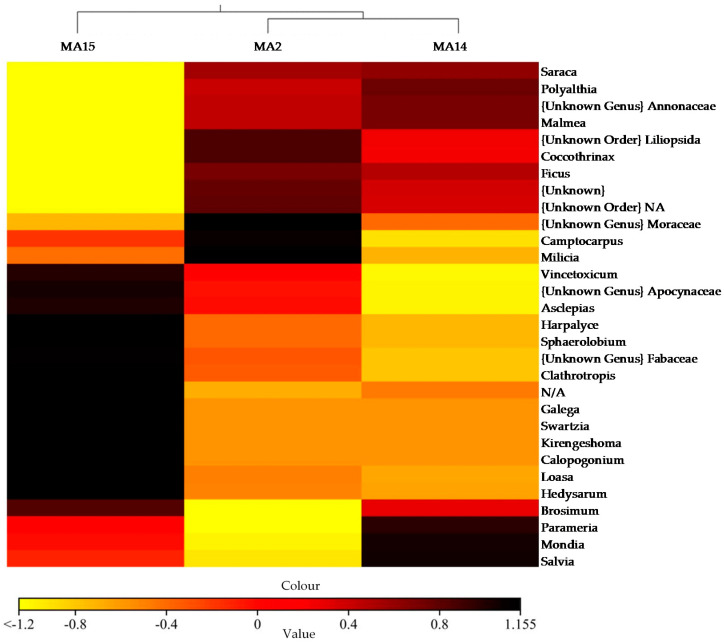
Heatmap with dendrogram at the genus level using a gradient heatmap (over 1% of the plant diversity).

Regarding composition level, heatmap examination revealed a remarkable interindividual variability in the plant communities’ consumption. The 30 most abundant genera were used in hierarchical clustering to evaluate the relationships between three samples of stump-tailed macaques using weighted pair clustering based on Bray–Curtis measurements (Figure 8). The value of genera consumed by these stump-tailed macaques was shown by color in the heatmap. The darker the red color, the more predominant the genus was consumed.

## 4. Discussion

This study is the first to report on the diversity of plants consumed by wild *M. arctoides* in the Malaysia–Thailand border region using DNA metabarcoding of tRNL chloroplast genes. The number of fecal samples in this study was low, primarily because this species is difficult to find in its natural habitat. It has been estimated that the home range of *M. arctoides* is several square kilometers [23]. Stump-tailed macaques are found below 1500 m in subtropical evergreen forests and between 1800 and 2500 m in tropical evergreen rainforests [24]. In a preliminary observation of *M. arctoides* in PSP, Wang Kelian revealed that these macaque groups were extremely sensitive to human presence and difficult to observe [3]. Even if the number of fecal samples is low (*n* = 3), it still provides a good idea of what stump-tailed macaques eat and solid information on estimation of the varieties of their diet intake. This is supported in a study by Srivathsan et al. [17] who identified the effectiveness of a metabarcoding tRNL approach for diet analysis using two fecal samples of a leaf-feeding monkey, *Pygathrix nemaeus* (red-shanked doucs langurs). Analyses of plant DNA in feces of *Gorilla gorilla* (gorilla) and *Colobus guereza* (white colobus) (*n* = 4 per species) provide a basic evaluation of minimum primate dietary diversity [25]. Non-invasive fresh fecal sampling was implied in the present study without capturing, handling, and restraining the individual stump-tailed macaques. This type of sampling is valuable because it can provide ecological and biological information without direct observation [26]. The impact of sample size has long been a challenge beyond the scope of DNA barcoding. Austerlitz et al. [27] found that study results improved with increased sample size when comparing phylogenetic and statistical classification methods for DNA barcoding. In addition, haplotype richness increased with sample size based on a non-parametric resampling approach [28]. In contrast, Liu et al. [29] proposed that sampling a single individual in a population was sufficient in a plant barcoding study of the *Taxus* species. Similarly, our study provides a preliminary evaluation and the potential means for assessing the diet of wild *M. arctoides* in PSP despite larger sample numbers. Nevertheless, high sample numbers are required when comparing diets between populations, geographic areas, and seasons. The dissimilar populations within a species may exhibit widely divergent diets, especially in species with a large geographic distribution [30].

Perlis State Park (PSP) which is situated in northernmost state in Peninsular Malaysia, Perlis, is the only semi-deciduous forest in the country. The uniqueness of this semi-deciduous forest has led to the gazettement of Perlis State Park as a protected biodiversity conservation area in Malaysia [31]. A semi-deciduous forest with most areas consisting of limestone hill forests and a small portion of granite-based parent material is exhibited in Perlis State Park (PSP) [32]. We identified a wide diversity of plant taxa consumed by the wild stump-tailed macaque in PSP. Among the representative macaque samples, we successfully identified 145 plant species. The most abundant species were yellow saraca, *Saraca thaipingensis* (11.70%), common fig, *Ficus carica* (9.33%), aramata, *Clathrotropis brachypetala* (5.90%), sea fig, *Ficus superba* (5.44%), and envireira, *Malmea dielsiana* (1.70%). Alpha diversity analysis in Table 2 suggested the *M. artoides* has the wider diet from PSP that shape the greater plant diversity. The vast diversity of the plant species has linked to the quality of habitat. According to Figure 6, the Venn diagram showed that individuals of MA2 and MA14 shared the highest number of OTUs which is 16,295. Similarly, PCoA analysis reveals the close relationship between MA2 and MA14. However, the diversity *t*-test shows significant differences between these two individuals (*p* < 0.05). Regarding composition level as shown in Figure 8, heatmap examination revealed a remarkable interindividual variability in the plant communities’ composition. Each sample harbored unique plant diet for abundance and richness of species. However, the type of forest in PSP is dissimilar due to the unique climate of Perlis. During dry season, fleshy fruit availability decreased, and thus favored stump-tailed macaques to become seed predators and flower eaters. Fleshy fruits are less abundant in semideciduous forests than in the wet forests [33]. Macaques are also frugivorous, though their diet comprises seeds, leaves, flowers, and tree barks although another macaque, the crab-eating macaque (*M. fascicularis*), persists on a diet of invertebrates and small vertebrates [9]. In older publications, the free-ranging *M. arctoides* has been reported to consume vegetal matter such as leaves, seeds, roots, flowers, and tree bark [34]. Surprisingly, this macaque has been observed occasionally hunting insects, water snails, reptiles, and birds [35]. Another primate species, *Propithecus tattersalli* (lemur), was exhibited to demonstrate remarkable dietary diversity with at least 130 plant species belonging to 80 genera and 49 different families during the dry season in northeastern Madagascar [36].

*Saraca thaipingensis* (yellow saraca) represent the highest number of plant taxa detected in all samples of *M. arctoides*. This is a species of plant in the Fabaceae family which is native to Peninsular Malaysia and is abundant along the river, which represents a typical element of the Malayan flora [37]. To the best of our knowledge, this is the first study to identify *S. thaipingensis* in the diet of a primate species, especially *M. arctoides*. This finding strongly suggests that *S. thaipingensis* represents the core diet of *M. arctoides* in PSP. The fig trees, *F. carica* and *F. superba*, are two species of the genus *Ficus* that also contribute to the diet of this macaque. *F. carica* is commonly known as the fig tree and is native to the Middle East and West Asia, whereas *F. superba* can be found in various parts of Southeast Asia including Peninsular Malaysia [38,39]. However, another species of fig tree is also distributed throughout Southeast Asia and comprises most of the macaque diet. Cercopithecidae is the major fig-eating family among mammals [40]. This is supported in a study by Hambali et al. [41] which identified *F. microcarpa* (curtain fig) as a favorite staple of the long-tailed macaque in Kuala Selangor Nature Park, Malaysia. Another related well-distributed fig tree species is *F. variegate*. Nila et al. [11] reported that *F. variegate* is one of the natural food preferences consumed by *M. fascicularis* in Telaga Warna, Bogor, West Java Indonesia. A study conducted by Ruslin et al. [12] also concluded that *F. variegate* was the most consumed food for *M. fascicularis* in Malaysia. *F. glaberrima* and *F. religiosa* are other fig species belonging to the Moraceae family and are part of the diet of another macaque group, *M. assamensis*, in Northern Thailand [42]. Fig fruits are higher in digestible carbohydrates and lower in fiber content compared to other forest fruit species [43]. Conversely, the Assamese macaque, residing in limestone habitats of Nonggang, China, consumes the bamboo species, *Indocalamus calcicolus*, as its main dietary constituent [44]. Most of these studies used direct diet observation and micro-histological analyses of fecal samples. There are no reports using fecal DNA metabarcoding on the macaque diet.

In our study, the diet composition of *M. arctoides* identified two species that are not known as Malaysia trees. Aramata tree, *C. brachypetala*, belongs to the Fabaceae family, whereas envireira, *M. dielsiana*, is from the Annonaceae family. Both *C. brachypetala* and *M. dielsiana* originate in Colombia and Peru, respectively [45,46]. These two species are currently not searchable in the plant database, so they were considered to be closely related relatives of these species. According to Cardoso et al. [47], the genus *Clathrotropis* belongs to the tribe *Ormosia* based on a phylogenetic analysis of plastid matK and tRNL intron data. The tribe *Ormosia* belongs to the plant family Fabaceae and primarily grows in tropical regions of the Americas, North Australia, and Southeast Asia [48]. There are 14 species of tribe *Ormosia* found in Malaysia. Most of these species are endemic to Peninsular Malaysia including *Ormosia polita*, *Ormosia grandistipulata*, and *Ormosia bancana* [49,50]. *Malmea* is a plant genus of the Annonaceae family. Malmeoideae is a new subfamily of the pantropical flowering family, *Annonaceae* and *Malmea* represents one of the new tribes classified in this subfamily [51]. A molecular phylogenetics study of *M. dielsiana* has been performed using plant DNA barcoding [52], and there is no report of this species being found in Malaysia. However, *Maasia sumatrana* is a closely related species found in Malaysia. *Maasia* is another new tribe of the Malmeoideae subfamily, and this tribe has been introduced into this subfamily from the *Polyalthia* species [51,53]. The work presented here indicates that the database of the Fabaceae and Annonaceae Malaysian tree families is limited. Therefore, tRNL sequencing results in the detection of closely related species are found in other countries.

The tRNL intron has been widely evaluated as a standard sequence that provides superior performance in identifying plant species and for phylogenetic studies based on data available in Gene Bank [16,19,25]. The tRNL sequences were successfully amplified in our study, and the number of sequences obtained was sufficient to evaluate the plant diet of wild *M. arctoides*. Valentini et al. [54] proposed the tRNL DNA metabarcoding approach as an indirect method for assessing the diet of a wide range of phytophagous species on a large scale based on the availability of plant databases. However, the lack of a good reference database limits the studies using DNA metabarcoding, particularly when the diet of the study species is not well characterized. In addition, chloroplast abundance is variable among different plant species [55]. Variability in different parts of the leaf depends on sequence specificity of the primers, so there is a chance that an unknown plant species will not be detected because of primer mis-priming [56]. It is important to note that our lack of data on home range, resource availability, and resource abundance may not be confounding variables, based on the results of other studies of wild macaques. Nevertheless, our study succeeded in identifying plant diversity as preliminary understanding of the *M. arctoides* diet using the tRNL DNA metabarcoding approach. Consequently, additional studies will be needed to increase the resolution power of the method using additional DNA barcodes with a more restricted taxonomic coverage of the Malaysian trees.

## 5. Conclusions

This study examined the utility of the tRNL DNA metabarcoding approach to identify plant diversity in the diet of wild *M. arctoides*. This non-invasive approach provides a preliminary understanding of the *M. arctoides* diet in PSP. Overall, our results suggest that the identification of plant taxa up to the species level is generally achievable based on non-coding regions of chloroplast DNA. The results of this study are preliminary because of a limited number of samples, home range, and the challenge of obtaining them in a natural habitat. With further optimisation, this method could provide a basic assessment of *M. arctoides* dietary diversity. This may serve as a basis for future studies regarding the conservation and management of this species. This non-invasive approach is especially important for the study of the diet of endangered primate species for which it is almost impossible to obtain observational data. In the future, to fully understand the dietary diversity of the wild *M. arctoides*, further studies focusing on the population and individuals by different demography, behavior, age, and sex are needed.

## Figures and Tables

**Table 1:** Total number of plants identified by tRNL DNA metabarcoding.

Taxonomic Level	*M. arctoides* tRNL
Order	29
Family	46
Genus	124
Species	145

**Table 2:** Alpha diversity indices: Simpson, Shannon, Evenness, and Chao-1.

Sample	MA15	MA2	MA14
Simpson_1-D	0.9937	0.9711	0.9644
Shannon_H	8.106	7.387	7.288
Evenness_e^H/S	0.1417	0.05611	0.04545
Chao-1	25,210	40,080	38,610

Simpson_1-D: Simpson diversity index; Shannon_H: Shannon diversity index; Evenness e^H/S: Species evenness Index; Chao-1: Chao-1 richness estimator.
